# Enzymatic activity of human immunodeficiency virus type 1 protease in crowded solutions

**DOI:** 10.1007/s00249-019-01392-1

**Published:** 2019-08-28

**Authors:** Ksenia Maximova, Jakub Wojtczak, Joanna Trylska

**Affiliations:** 1 Centre of New Technologies, University of Warsaw, Banacha 2C, 02-097 Warsaw, Poland; 2 Faculty of Biology, University of Warsaw, Miecznikowa 1, 02-096 Warsaw, Poland

**Keywords:** Enzymatic reactions, Human immunodeficiency virus type 1 protease (HIV-1 PR), Crowded environment, Kinetic parameters, Fluorescence spectroscopy, Polyethylene glycol

## Abstract

Cells are crowded with various macromolecules and metabolites, which affect biochemical reactions in many ways, from the diffusion of substrates to catalytic activities of enzymes. We herein investigated the proteolytic activity of the human immunodeficiency virus type 1 protease (HIV-1 PR) under non-crowded and crowded conditions. The latter environment was mimicked with various (poly)ethylene glycol molecules as crowding agents. We found that these crowding agents affect the kinetic parameters of the HIV-1 PR catalyzed reaction by increasing the Michaelis–Menten constant and decreasing the maximum velocity. The influence of crowding was concentration dependent. We explain this effect by the dynamics of the HIV-1 PR flexible flaps that cover the peptide substrate binding site and are crucial for enzyme activity, and by a possibly slower substrate–enzyme association time in the crowded conditions.

**Electronic supplementary material:**

The online version of this article (10.1007/s00249-019-01392-1) contains supplementary material, which is available to authorized users.

## Introduction

Cells typically contain between 200 and 400 g/L of macro- and small molecules such as proteins, nucleic acids, ribosomes, metabolites, and lipids (Zimmerman and Trach 1991). Such a crowded environment could affect many aspects of biochemical reactions occurring in vivo (Minton 2001; Zhou et al. 2008). For instance, crowding may influence the diffusion of molecules (Szymański et al. 2006; Yu et al. 2016), enzyme folding, internal dynamics, functional conformations, catalytic activity, product release, and many other events that are still not well understood (Minton 2001; Hall and Minton 2003; Długosz and Trylska 2011; Miklos et al. 2011; Christiansen et al. 2013; Kuznetsova et al. 2014). Therefore, the kinetic constants of enzymatic reactions estimated in dilute (non-crowded) conditions may differ by many orders of magnitude from the ones in the living cell. To reflect in vivo enzymatic reactions, the experiments have to be performed at least under crowded conditions. We herein show that crowded conditions affect the kinetic parameters of the reaction catalyzed by the human immunodeficiency virus type 1 protease (HIV-1 PR). HIV-1 PR is an enzyme essential for the viral replication and is a drug target in HIV-1 infections (Clercq 2007).

**Fig. 1:**
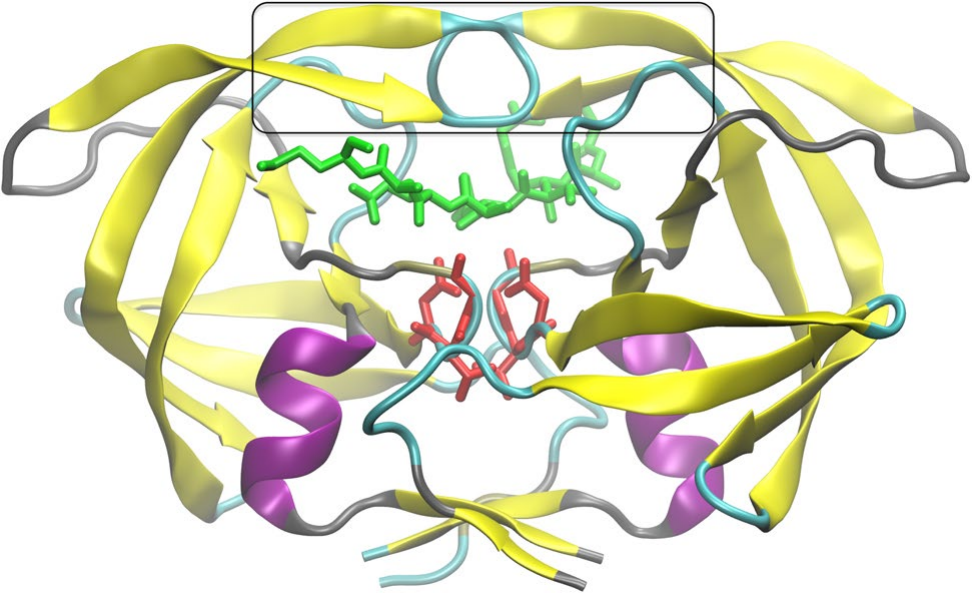
Tertiary structure of the HIV-1 PR backbone (PDB code: 1F7A (Prabu-Jeyabalan et al. 2000)) with β-sheets in yellow and helices in magenta. The active site triad is shown in red and the substrate in green. The frame schematically marks the flap region

HIV-1 PR is an aspartic protease (Fig. 1). It exists as a homodimer, with 99 amino acids and a catalytic center composed of D25, T26, and G27 in each monomer, where both D25 aspartic acids play a role in catalysis. HIV-1 PR dimer has two flaps closing over the substrate (Tóth and Borics 2006; Chang et al. 2007; Trylska et al. 2007; Miao et al. 2018), whose mobility is crucial for both substrate association and binding (since the flap tips can be as far apart as 40 Å) and for assuring substrate functional fluctuations in the binding site (Scott and Schiffer 2000; Piana et al. 2002; Tozzini et al. 2007; Karthik and Senapati 2011). Molecular dynamics simulations showed that crowder molecules influence the conformational dynamics of these flaps (Minh et al. 2006; Qin et al. 2010), as well as HIV-1 PR interactions with ligands (Kang et al. 2011). It was also shown for other dimers that protein shape affects the interactions with crowders (Guseman et al. 2018).

In this study we performed fluorescence-detected enzymatic assays in non-crowded and crowded solutions to estimate the impact of crowding on the enzymatic activity of HIV-1 PR. For the hydrolysis we selected a peptide substrate (Matayoshi et al. 1990; Windsor et al. 2015) that was labelled to observe changes in the Förster resonance energy transfer (FRET) upon cleavage. The FRET substrate was manually synthesized using the Fmoc solid-phase peptide synthesis method. Crowding effects were mimicked with either ethylene glycol (EG) or polyethylene glycol (PEG) molecules of different masses (0.06 kDa EG, 0.6 kDa PEG600, and 6 kDa PEG6000) and concentrations. To reach the concentrations mimicking the cellular conditions (Zimmerman and Trach 1991), we increased the crowder concentration up to 300 g/L.

## Materials and methods

### Reagents

HIV-1 protease (HIV-1 PR) was purchased from ProSpec-Tany TechnoGene Ltd. and all other chemicals from Sigma Aldrich. 365 nM HIV-1 PR stock solution was prepared in 100 mM sodium acetate buffer at pH 4.7 at 37 °C with 1 M NaCl, 1 mM EDTA and 1 g/L BSA and stored at − 80 °C.

### Substrate synthesis

The HIV-1 PR FRET substrate [Arg-Glu(EDANS)-Ser-Gln-Asn-Tyr-Pro-Ile-Val-Gln-Lys(DABCYL)-Arg-NH_2_, Figure S4] was synthesized by the solid-phase method using Fmoc-chemistry [EDANS stands for a 5-((2-aminoethyl)amino)naphthalene-1-sulfonic acid and DABCYL for 4-((4-(dimethylamino)phenyl)azo) benzoic acid]. Tenta Gel resin (90 µM mesh, 0.24 mmol/g loading) was used as a solid support. Briefly, 100 mg of the resin was swelled in 2 mL of DMF for 20 min. Then Fmoc-deprotection step was performed by 2 mL of 20% piperidine in DMF, two times for 15 min, and the resin was washed by DMF. Then 3 eq. of the following: Fmoc-AA-COOH was coupled by 3 eq. of HATU, 3 eq. of HOAt, 3 eq. of colidine, and 0.03 eq. of DMAP. The reaction mixture was stirred for 1.5 h at room temperature under argon. The coupling reaction was repeated one more time. Next, the reaction mixture was washed by DMF. After completing the synthesis, the peptide was cleaved from the resin and protecting groups (Boc, tBu) were removed using the TFA/H_2_O/TIPS mixture [74/2/1 (v/v/v)] and stirring the reaction mixture for 3 h. The final solution was filtered to cold ether. The precipitate from ether was centrifuged and washed by ether. The purity of the peptide was checked on a reverse-phase HPLC SYKAM equipped with a KNAUER C18 column (8 × 250 mm) and a UV–Vis detector. A linear gradient from 75 to 25% water to acetonitrile with 0.1% TFA within 30 min at the flow rate 1.5 mL/min was applied. The analyzed peptide was monitored at 267 nm. The collected fraction was lyophilized. The purity of the synthesized peptide was confirmed by HPLC (Figure S5) and mass spectrometry (Figure S6). Formula: C_92_H_133_N_27_O_23_S; calculated [M]^+^ = 2016.29, found [M]^+^ = 2015.7650.

### Fluorogenic assay

The cleavage of the FRET peptide substrate by HIV-1 PR increases fluorescence emission of the EDANS donor. EDANS is no longer quenched by DABCYL because their mutual distance increases after hydrolysis. Fluorescence was measured on a microplate reader from BioTek (Winooski, United States). The excitation wavelength was 340 nm and the emission wavelength was 490 nm. The HIV-1 PR FRET substrate was prepared in DMSO as 10 mM solution and stored at − 80 °C. All experiments were performed without crowding or in solutions crowded by EG, PEG600, PEG6000 or BSA. The concentrations of the crowding agents were 100, 200 and 300 g/L. All experiments with and without crowders were performed in 100 mM sodium acetate buffer at pH 4.7 at 37 °C with 1 M NaCl, 1 mM EDTA, 1 g/L BSA, 1 mM DTT and 15% DMSO in volume of 100 µL per well. The fluorescence data were fitted using GraphPad. All experiments were performed as independent triplicates. The averages with their standard deviations are shown as results.

#### EDANS calibration

The 1000 µM EDANS acid solution was twofold serial diluted with final EDANS concentrations of 1000, 500, 250, 125, 62.5 and 31.25 nM in an assay buffer with and without crowders.

#### HIV-1 PR catalyzed hydrolysis

The hydrolysis of HIV-1 PR FRET substrate was performed by 4.4 nM HIV-1 PR. The substrate concentrations were 120, 100, 75, 35 and 15 µM. The enzyme solution was added immediately before each measurement. The fluorescence signals were recorded every 30 s for 30 min.

## Results

**Fig. 2:**
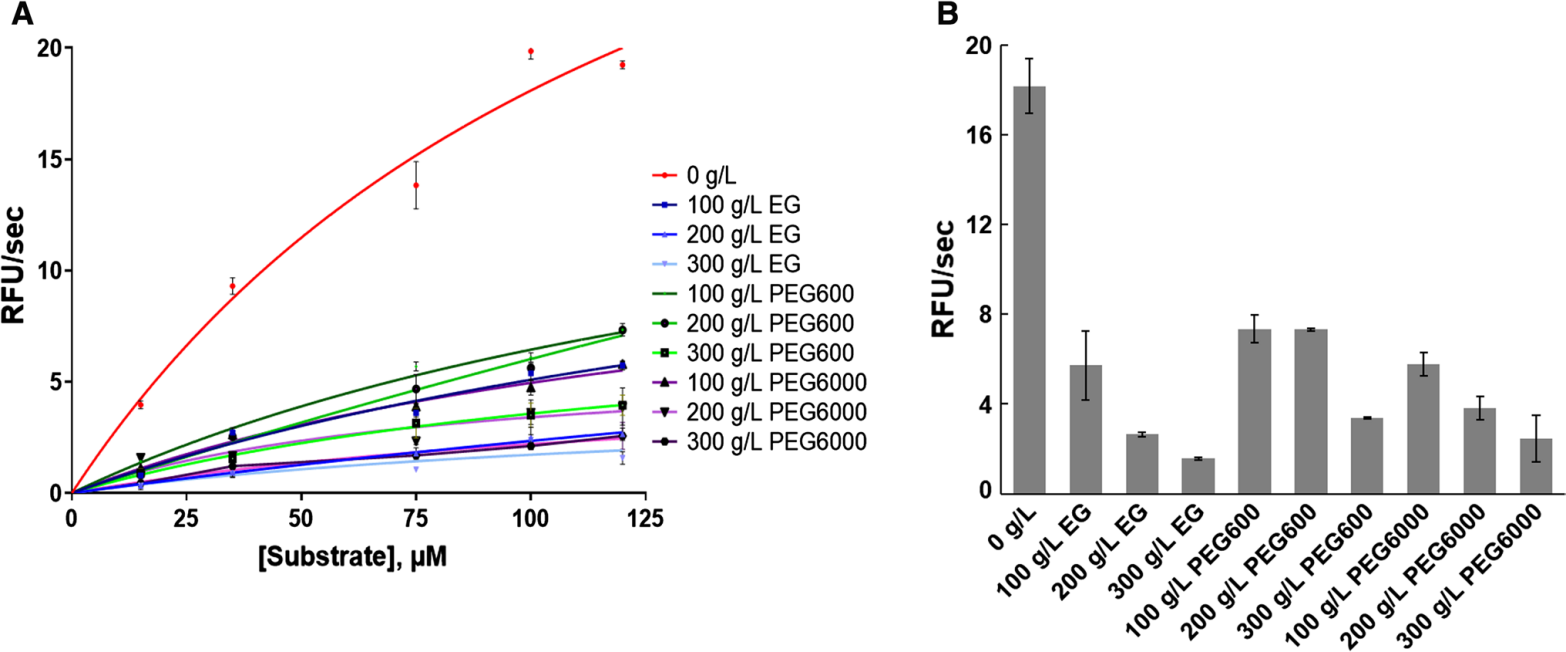
**a** Fluorescence changes in relative fluorescence units (RFU) per second during the HIV-1 PR catalyzed hydrolysis of the FRET substrate in dilute and crowded solutions. **b** Comparison of initial velocities for the hydrolysis of 120 µM of the substrate catalysed by HIV-1 PR. Statistical significance of the differences between the reaction in non-crowded and crowded solutions was determined by the ANOVA test and is equal to *p* < 0.0001 for all crowder types

The results of our enzymatic assays clearly showed that the presence of crowding agents influences the reaction rates (Fig. 2). The substrate conversion to the products significantly decreased even in the presence of the smallest EG crowder and already at its lowest concentration of 100 g/L (Fig. 2a—dark blue line). Moreover, the influence of crowding was concentration dependent; the HIV-1 PR activity declined along with the increased crowder concentration (Fig. 2b).

Additional control experiments monitoring the emission of the buffer, substrate, and product in crowded solutions confirmed that the decrease of the fluorescence signal was due to the influence of crowding on the catalyzed reaction and not due to quenching of the fluorescence signal by the crowding itself. For instance, the fluorescence signals for the buffer, as well as for EG, PEG600 and PEG6000 at different concentrations (100, 200 and 300 g/L) were negligible (Figure S1). However, the bovine serum albumin (BSA), which is often used as the crowding agent in experiments, gave significant emission by itself. This is why, in our studies, we excluded BSA as a crowder.

Moreover, we checked if crowding influenced the emission of free EDANS (Figure S2). Experiments have shown that the EDANS emission changes between the non-crowded and crowded solutions were not statistically significant. However, this information does not help explain the observed decrease in the fluorescence signal during the HIV-1 PR catalyzed reaction in the crowded solutions (Fig. 2). Additionally, we checked the inner filter effect (Liu et al. 1999). The emission of EDANS in the presence of the substrate did not change significantly in the crowded solutions and was not accounted for further determination of the kinetic parameters of the HIV-1 PR catalytic activity (Figure S3).

The Michaelis–Menten constants are presented in Table 1 and suggest that crowding influences the kinetic parameters, which is also in accord with the fluorescence changes shown in Fig. 2. However, note that the data for higher crowder concentrations of 300 g/L EG, as well as of 200 and 300 g/L PEGs, did not follow the Michaelis–Menten curve, so they were not used to fit the kinetic constants. Data for these higher concentrations are shown in Fig. 2 only for qualitative comparison. Nevertheless, for concentrations of 100 and 200 g/L of EG, as well as for 100 g/L of PEG600 and PEG6000, the kinetic constants were estimated and clearly showed an increase in *K*_m_. Importantly, all crowders significantly decreased *v*_max_. For example, *v*_max_ was equal to 66 RFU/s for the non-crowded solution, whereas *v*_max_ decreased sixfold in the solution crowded by 100 g/L of EG. As shown in Fig. 2, in general, higher crowder concentrations decreased the activity of HIV-1 PR to a larger extent.

**Table 1:** Kinetic constants of the HIV-1 PR catalyzed hydrolysis of a peptide substrate without and with crowders

Crowding	0 g/L	EG 100 g/L	EG 200 g/L	PEG600 100 g/L	PEG6000 100 g/L
*V*_max_ (RFU/s)	66 ± 4	11 ± 1	16 ± 6	19 ± 5	13 ± 4
*K*_m_ (µM)	25 ± 5	86 ± 6	498 ± 221	189 ± 70	158 ± 60

## Discussion

Our fluorescence assays show that the catalytic activity of HIV-1 PR decreases in solutions that include either EG or PEG molecules as crowding agents. The range of the effect depends rather on the crowder concentration than their mass or size, even though EG is only 60 Da in mass and 3.5 Å in length, and the used PEGs are one and two orders of magnitude larger. Overall, many effects could contribute to the observed concentration dependent decrease in HIV-1 PR activity in the presence of EG and PEG molecules.

These results could be explained by the previously described effects of crowding on the internal dynamics of HIV-1 PR. With molecular dynamics simulations we have shown that spherical crowding agents affect the internal dynamics of the HIV-1 PR flaps (Minh et al. 2006). HIV-1 PR has a pair of flexible flaps (Fig. 1), which need to first open and next close over the peptide substrate for its proper accommodation in the binding site (Tóth and Borics 2006; Trylska et al. 2007). Therefore, the opening and closing of the flaps is involved in the enzymatic mechanism of this protease. The frequency of flap opening was shown to be suppressed at highly crowded environment (Minh et al. 2006; Qin et al. 2010), which may contribute to reducing the HIV-1 PR enzymatic activity in vivo, since the association of a substrate was suggested to be controlled by a rare event such as flap opening (Katoh et al. 2003). Also, the observed cooperative fluctuations of free and substrate-bound protease (Kurt et al. 2003) are probably affected by the presence of crowders at high enough concentrations. Note, that such effect of shifting the equilibrium to the closed states was also found for many other proteins that have distinct open-closed conformations determined experimentally (Dong et al. 2010).

In addition, Brownian dynamics simulations have shown that protein crowders slowed down the HIV-1 PR–ligand association times by two to fourfold in comparison with non-crowded systems (Kang et al. 2011). The observed effect was not only due to excluded volume but also crowder-ligand interactions. The initial stage of the reaction requires the formation of a stable substrate–enzyme complex, which is a multi-step process in itself. The fact that crowding was observed to slow down diffusion of the substrate and its association with HIV-1 PR (Kang et al. 2011), may also contribute to the observed decrease in the overall catalytic activity of HIV-1 PR.

Electrostatic interactions may also play a role. The FRET substrate has a total net charge of + 2e and an unbound HIV-1 PR dimer at least + 4e (considering standard protonation states of amino acids at pH 7 but the activity of HIV-1 PR was measured at pH 4.7). PEG molecules are hydrophilic and thus may influence the substrate association times not only due to occupying space but also due to EG or PEG–substrate interactions, as well as PEG–solvent interactions (PEG presence changes the solvent properties).

Therefore, overall many effects could contribute to the observed concentration dependent decreased activity of HIV-1 PR in the presence of EG and PEG molecules, and they are difficult to quantify. One aspect is that the HIV-1 PR enzyme is highly intrinsically dynamic, another one that the crowd affects enzyme–substrate interactions, as well as the diffusion of substrate toward the enzyme. Since HIV-1 PR has a pair of flexible flaps these effects could be even more difficult to assess than for other enzymes.

However, since HIV-1 genome mutates and resistance to currently available drugs targeting HIV-1 PR is a known issue, we believe that it is important to test the HIV-1 PR catalyzed reactions and the effect of any newly designed inhibitors in the presence of macromolecular crowders, and not only in dilute buffer solutions.

## Electronic supplementary material

Below is the link to the electronic supplementary material.

Supplementary file1 (PDF 206 kb)
