# Supplementary material for: Enzymatic activity of human immunodeficiency virus type 1 protease in crowded solutions
Source: Eur Biophys J. 2019 Aug 28;48(7):685–9. doi: 10.1007/s00249-019-01392-1 (PMC6742607; doi:10.1007/s00249-019-01392-1)
Supplement: Supplementary file 1 — Supplementary file1 (PDF 206 kb) [file 249_2019_1392_MOESM1_ESM.pdf]

Supporting Information

Enzymatic activity of human immunodeficiency virus type 1 protease in crowded solutions

Ksenia Maximova[1,*], Jakub Wojtczak[1,2] and Joanna Trylska[1,*]

[1]Centre of New Technologies University of Warsaw, Banacha 2C, 02-097 Warsaw, Poland

[2]Faculty of Biology, University of Warsaw, Miecznikowa 1, 02-096 Warszawa, Poland

*e-mail: k.maximova@cent.uw.edu.pl and joanna@cent.uw.edu.pl

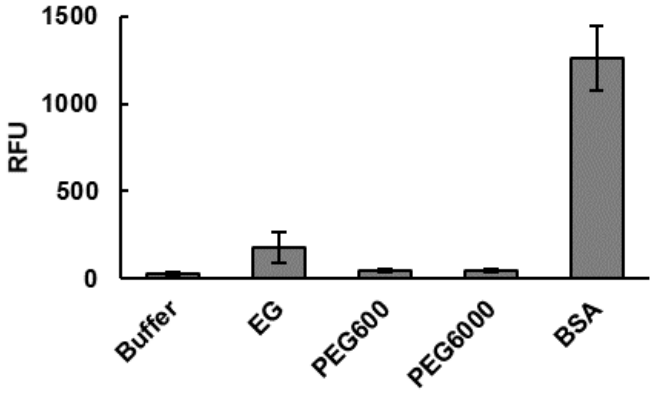

Figure S1. Fluorescence signals in relative fluorescence units (RFU) for the buffer without or with 100 g/L of EG, PEG600, PEG6000 or BSA.

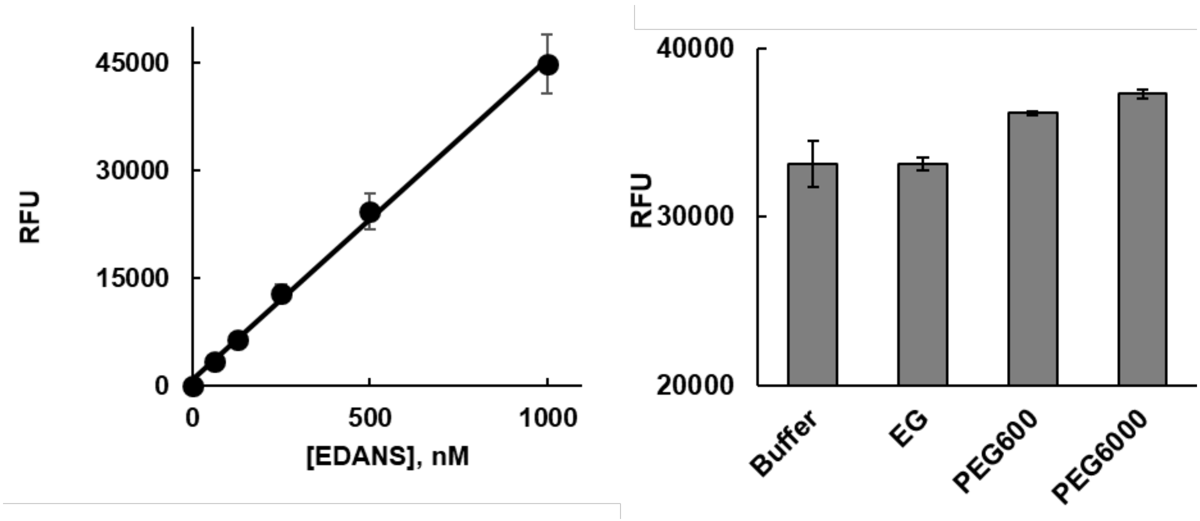

Figure S2. *Left:* EDANS emission in the buffer as a function of the EDANS concentration *Right:* The comparison of the EDANS signal in the buffer and in 100 g/L crowded solutions. Statistical significance of the differences between the EDANS emission in buffer and tested crowders was determined by ANOVA test and was not significant.

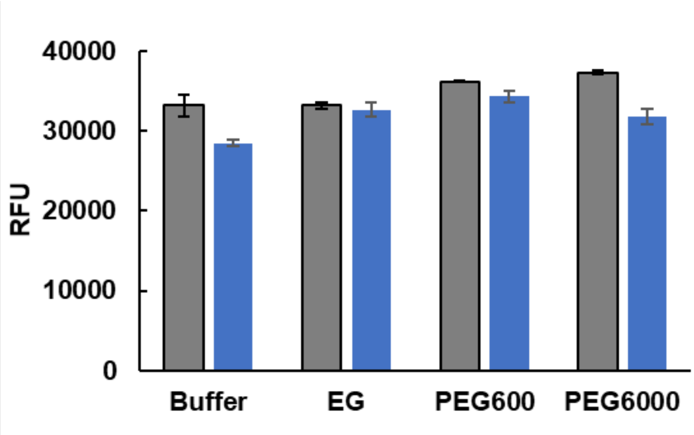

Figure S3. Fluorescence emission of EDANS (grey bars) and EDANS with 120 µM substrate (blue bars), in the buffer without or with 100 g/L EG, PEG600 or PEG6000. Statistical significance of the differences between the EDANS emission in the buffer and tested crowders was determined by ANOVA test and was not significant.

RESQNYPIVQKR

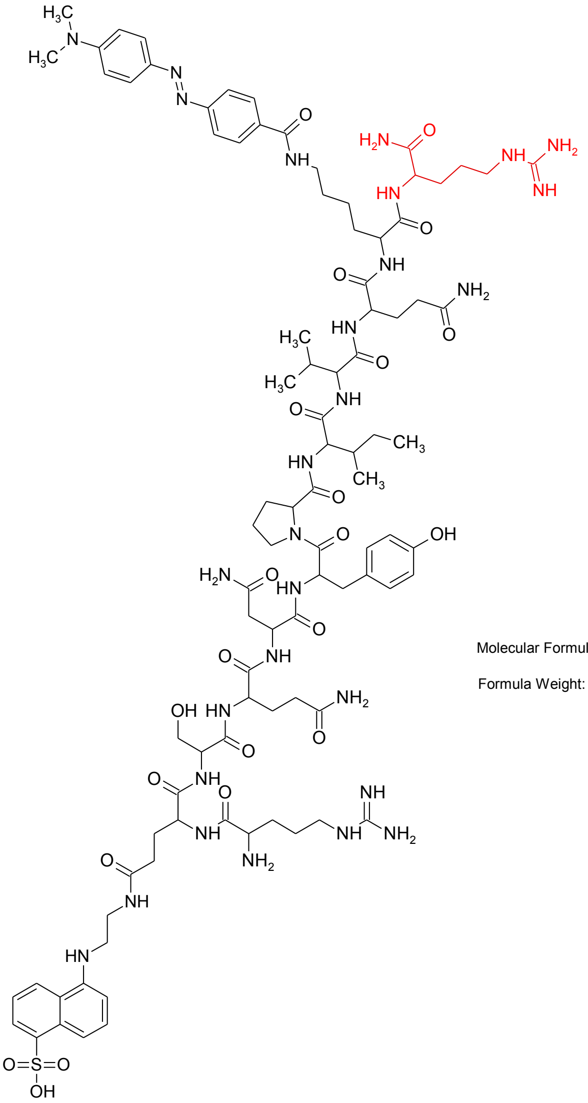

Molecular Formula: $C_{92}H_{134}N_{28}O_{22}S$

Formula Weight: 2016.28776

Figure S4. The chemical structure of the HIV-1-PR FRET substrate (Arg-Glu(EDANS)-Ser-Gln-Asn-Tyr-Pro-Ile-Val-Gln-Lys(DABCYL)-Arg-NH_2).

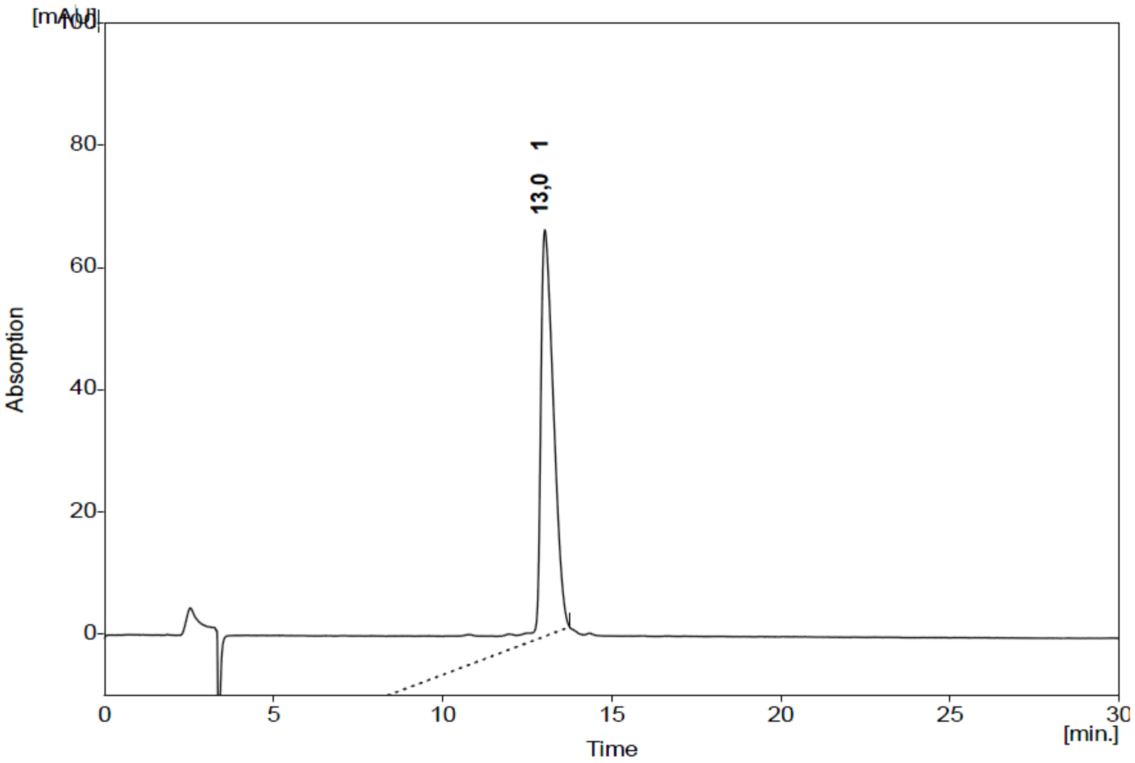

Figure S5. The HPLC chromatogram of the HIV-1-PR FRET substrate (Arg-Glu(EDANS)-Ser-Gln-Asn-Tyr-Pro-Ile-Val-Gln-Lys(DABCYL)-Arg-NH_2).

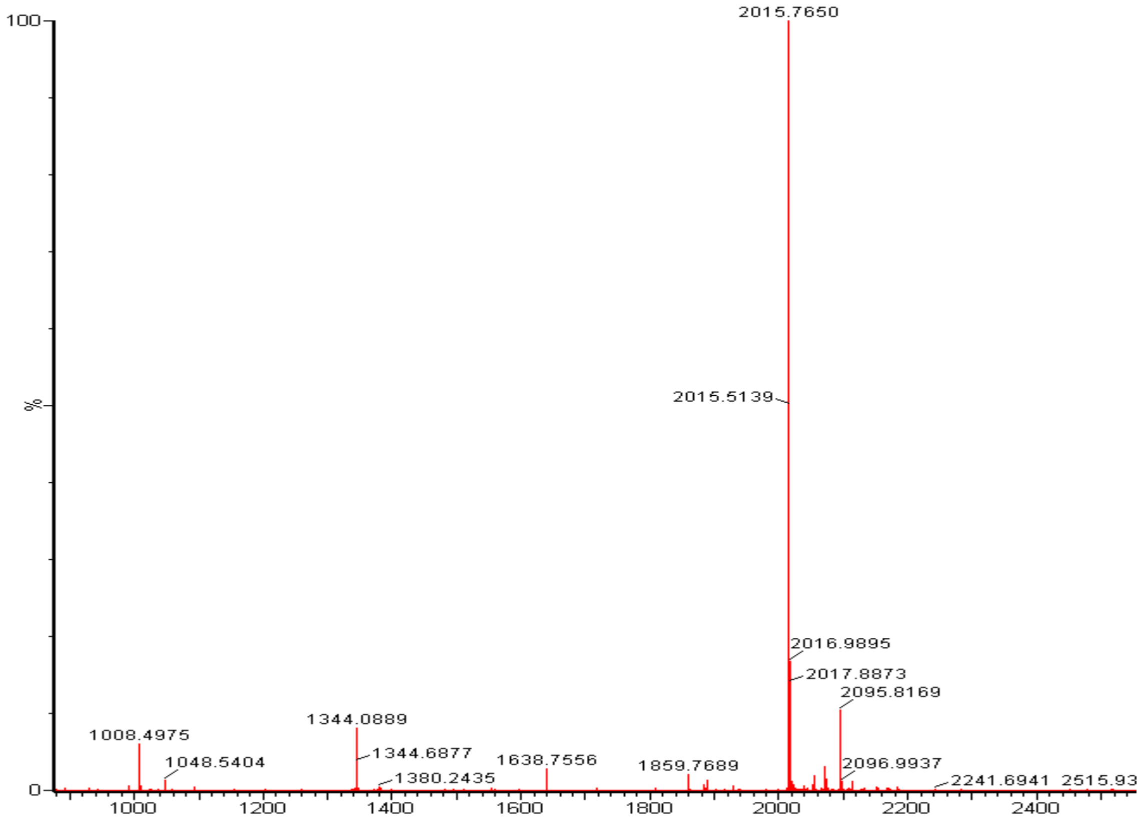

Figure S6. Mass spectrum of HIV-1-PR FRET substrate (Arg-Glu(EDANS)-Ser-Gln-Asn-Tyr-Pro-Ile-Val-Gln-Lys(DABCYL)-Arg-NH_2).
